# The Influence of Statin Therapy on Markers of Endothelial Dysfunction, Endocan and Endoglin, in Postmenopausal Women—A Prospective Study

**DOI:** 10.3390/biomedicines14091921

**Published:** 2026-08-27

**Authors:** Dunja Šojat, Marko Pirić, Maja Lukić, Vesna Horvat, Mario Šafer, Ivan Feldi, Tatjana Bačun, Aleksandar Kibel

**Affiliations:** 1 Department of Family Medicine, Faculty of Medicine, Josip Juraj Strossmayer University of Osijek, 31000 Osijek, Croatia; dunja.sojat@gmail.com (D.Š.);; 2 Health Center of Osijek-Baranja County, 31000 Osijek, Croatia; 3 Department of Medical Chemistry, Biochemistry and Clinical Chemistry, Faculty of Medicine, Josip Juraj Strossmayer University of Osijek, 31000 Osijek, Croatia; 4 Clinical Institute of Laboratory Diagnostics, University Hospital Centre Osijek, 31000 Osijek, Croatia; 5 Medical-Biochemical Laboratory Vesna Horvat, 31000 Osijek, Croatia; 6 Department of Internal Medicine, General Hospital Virovitica, 33000 Virovitica, Croatia; mario.safer27@gmail.com; 7 Department of Internal Medicine, General County Hospital Našice, 31500 Našice, Croatia; ivanfeldi@gmail.com; 8 Department of Internal Medicine and History of Medicine, Faculty of Medicine Osijek, Josip Juraj Strossmayer University of Osijek, 31000 Osijek, Croatia; 9 Department of Clinical Medicine, Faculty of Dental Medicine and Health Osijek, Josip Juraj Strossmayer University of Osijek, 31000 Osijek, Croatia; 10 Division of Endocrinology, Department of Internal Medicine, University Hospital Centre Osijek, 31000 Osijek, Croatia; 11 Department of Physiology and Immunology, Faculty of Medicine, Josip Juraj Strossmayer University of Osijek, 31000 Osijek, Croatia; 12 International Medical Center Priora, 31210 Cepin, Croatia

**Keywords:** postmenopause, hydroxymethylglutaryl-CoA reductase inhibitors, endothelial dysfunction, endocan, endoglin

## Abstract

**Background:** Postmenopausal women experience an increased risk of cardiovascular diseases due to estrogen deficiency, which induces endothelial dysfunction. Statins primarily lower cardiovascular risk by altering lipid profiles, but they also exert non-lipid-modifying, pleiotropic effects on the vascular endothelium. This prospective observational study was aimed to evaluate and compare the impact of atorvastatin and rosuvastatin on plasma markers of endothelial dysfunction, endocan and endoglin, in postmenopausal women undergoing primary cardiovascular prevention. **Methods**: A total of 128 postmenopausal Croatian women (aged 45–70 years) with dyslipidemia were prospectively enrolled and assigned to two treatment groups receiving either atorvastatin or rosuvastatin therapy based on primary care clinical management. Cardiovascular risk was evaluated using the SCORE2 algorithm, and statin doses were titrated up to 16 weeks until target LDL-C values were achieved. Plasma concentrations of endocan and endoglin, alongside lipid profiles, were quantified via ELISA and laboratory analyses before and after the treatment period. **Results**: Atorvastatin and rosuvastatin therapies significantly decreased plasma concentrations of both endocan and endoglin (*p* < 0.001 for both groups). No statistically significant differences in the absolute changes in these biomarkers were observed between therapy groups. The reduction in endocan and endoglin levels occurred independently of whether the participants achieved their clinical target LDL-C values. While statin-induced reduction in endoglin was directly driven by the extent of LDL-C lowering, the decrease in endocan occurred independently of lipid changes, with both biomarker reductions being greatest in women with higher baseline levels regardless of statin type or dose. ROC curve analysis demonstrated that neither biomarker had significant clinical discriminatory ability to predict the attainment of target LDL-C levels. **Conclusions**: Atorvastatin and rosuvastatin treatment was associated with significant reductions in plasma endocan and endoglin concentrations in postmenopausal women receiving primary cardiovascular prevention.

## 1. Introduction

Postmenopause is defined as the phase beginning one year after the final menstrual cycle, marked by hormonal shifts due to a reduction in the quantity and activity of ovarian follicles. According to the World Health Organisation (WHO) definition, this phase is identified after a minimum of one year has elapsed since menstrual bleeding stopped, provided that other hormonal or organic reasons for the absence of menstruation have been ruled out. It constitutes a substantial portion of a woman’s life, given that the average age at menopause is 50 years, meaning women spend nearly one-third of their lives in the postmenopausal phase [1]. Approximately 10% of women experience early menopause (occurring between 40 and 45 years of age), whereas menopause occurring before 40 years of age is defined as premature menopause [2].

Despite considerable advancements in the diagnosis, prevention, and treatment of cardiovascular disease (CVD), it continues to be the primary cause of mortality worldwide for both genders [3,4]. CVD exhibits significant differences between the sexes in terms of clinical symptoms, outcomes, and underlying pathophysiological processes, making it more severe in women [5]. While premenopausal women generally face a lower cardiovascular risk than age-matched men, this protective advantage rapidly declines in postmenopause, with CVD incidence increasing up to six-fold [6,7].

Perimenopause represents the physiological transition leading to the final menstrual period, initiated by the onset of menstrual cycle length variability. Driven by the progressive depletion of ovarian follicular activity, this stage is characterized by pronounced fluctuations and an ultimate decline in circulating estrogen and progesterone levels. Although perimenopause lasts an average of 4 to 7 years, its duration demonstrates marked individual variation, extending up to 14 years [8]. Research indicates that estradiol levels start to decrease two years before the last menstrual cycle, whereas FSH levels rise [9].

Estrogen has numerous positive effects on the cardiovascular system and vascular endothelium. It facilitates vasodilation by interacting with it via prostacyclin, thromboxane, and nitric oxide (NO). Its positive impact is also evident in its ability to raise high-density lipoprotein cholesterol (HDL-C) levels and lower low-density lipoprotein cholesterol (LDL-C) and total cholesterol (TC) levels [10]. Additionally, estrogen positively influences coagulation and fibrinolysis by reducing factor VII, fibrinogen, and plasminogen activator inhibitor-1 (PAI-1) levels. It has beneficial metabolic effects, such as decreasing abdominal fat and enhancing glucose metabolism [11,12,13,14]. Many studies have demonstrated the anti-inflammatory and antioxidant properties of estrogens. Consequently, its deficiency during the postmenopausal period fosters systemic chronic inflammation and oxidative stress, which further impairs endothelial function and deteriorates cardiovascular health [10].

The rise in CVD cases and the increased likelihood of fatal outcomes following cardiovascular events during postmenopause are linked to rapid progression of atherosclerosis. This is mainly due to the reduction in estrogen levels in postmenopausal women, which leads to the loss of previously mentioned beneficial cardiovascular health factors [15]. Postmenopause is also characterized by proatherogenic shifts, including marked increases in apolipoprotein B (ApoB) levels—which often surpass those of men—and a functional impairment of HDL-C, which may lose its antiatherogenic capacity during this period [16,17,18].

Endothelial dysfunction, marked by heightened oxidative stress, elevated inflammation, and altered vasodilation, increases the likelihood of CVD in postmenopausal women [19]. The lack of estrogen diminishes the bioavailability of NO, resulting in compromised endothelial vasodilator function and increased vascular stiffness, whereas elevated oxidative stress hampers normal endothelial activity [20,21]. Postmenopausal women experience low-grade systemic inflammation and higher levels of proinflammatory cytokines, including tumor necrosis factor alpha (TNF-α), interleukin-6 (IL-6), and interleukin-1beta (IL-1β). Plasma levels of adhesion molecules such as intercellular adhesion molecule 1 (ICAM-1) and vascular cell adhesion molecule 1 (VCAM-1), which are indicators of endothelial dysfunction crucial for leukocyte migration and adhesion and the inflammatory response that promotes atherogenesis, are also elevated in the postmenopause [22,23,24].

Endocan plays a crucial role in the inflammatory response by influencing the proliferation, migration, and adhesion of inflammatory cells. It is involved in various other processes connected with inflammation, including enhanced vascular permeability and angiogenesis [25,26]. Present understanding indicates a link between high plasma endocan levels and systemic inflammation, autoimmune diseases, and CVD. Additionally, increased plasma endocan levels are thought to indicate endothelial dysfunction [25,27].

Furthermore, the expression of membrane endoglin in endothelial cells is important in the process of formation and progression of atherosclerosis, considering that endoglin is the basic component of the activation of the endothelial nitric-oxide synthase (eNOS) complex, important for vasodilatation of blood vessels and adequate functioning of the vascular endothelium [28]. Studies have demonstrated that hypercholesterolemia affects endothelial dysfunction by decreasing membrane endoglin expression, which in turn reduces eNOS activation and NO production. This reduction fosters inflammation and elevates soluble endoglin (sEng) levels. These findings highlight the link between diminished sEng expression and NO production prior to noticeable atherosclerotic changes, suggesting that membrane endoglin expression is vital for preventing endothelial dysfunction. Elevated sEng levels exacerbate endothelial dysfunction, contribute to arterial hypertension, and may act as an indicator of metabolic syndrome [29].

Many epidemiological studies and randomized controlled trials have repeatedly demonstrated a log-linear correlation between alterations in serum LDL-C levels and the likelihood of developing atherosclerotic cardiovascular disease (ASCVD) [30,31]. The accumulation of atherosclerotic plaque and modifications in its composition contribute to ASCVD; thus, medications that reduce LDL-C and other ApoB-containing lipoproteins are advised for both primary and secondary prevention of atherosclerotic cardiovascular incidents [32].

To decrease TC and LDL-C levels, 3-hydroxy-3-methylglutaryl-coenzyme A (HMG-CoA) reductase inhibitors, commonly known as statins, are recommended as the primary treatment option [33]. Statins primarily reduce cardiovascular risk by altering lipidogram parameters, particularly by lowering LDL-C levels. Additionally, they decrease cardiovascular risk via pleiotropic effects: influencing the stability of atherosclerotic plaques, inflammation, endothelial (dys)function, and thrombogenesis. The pleiotropic nature of statins is largely due to the inhibition of HMG-CoA reductase in liver cells, which subsequently inhibits isoprenoid synthesis. This inhibition prevents the post-translational modification of small intracellular proteins that bind guanosine-triphosphate from the Ras, Rho, and Rac kinase families. By affecting these intracellular proteins, statins regulate the cell cycle, smooth muscle cell migration, and the expression of plasminogen PAI-1 and eNOS, thereby altering atherogenesis and thrombogenesis. Statins exert anticoagulant effects by downregulating tissue factor and upregulating endothelial thrombomodulin, which together reduce thrombin generation. They are also associated with impaired fibrinogen cleavage and reduced activation of factors V and XIII, likely through modulation of factor V/activated factor V, the protein C pathway, and tissue factor pathway inhibitor activity. In addition, clinical and experimental studies indicate that statins have antiplatelet effects, characterized by inhibition of platelet activation, adhesion, and aggregation [34,35].

This study aimed to evaluate the impact of atorvastatin and rosuvastatin on the concentrations of plasma markers of endothelial dysfunction, endocan and endoglin, in postmenopausal women, in whom statin therapy was used for the purpose of primary cardiovascular prevention, since similar studies have not been conducted so far. Understanding the impact of these types of statin therapy on markers of endothelial function may enable a more personalized approach to cardiovascular risk reduction in postmenopausal women.

## 2. Materials and Methods

### 2.1. Study Design

The study was designed as a prospective observational study. A total of 128 postmenopausal women, aged up to 70 years, from 15 family medicine practices in the Osijek-Baranja County, Croatia, participated between January 2024 and June 2025. The participants were women who visited their family doctor for a preventive examination and who met the inclusion criteria. Statin therapy (atorvastatin or rosuvastatin) was prescribed by their family physicians according to standard cardiovascular risk prevention guidelines. The study analyzed the levels of endocan and endoglin in patients before and after the initiation of routine statin therapy, without any investigator-assigned interventions or alterations to the patients’ standard care. Therefore, a clinical trial registration number is not applicable to this study.

Each participant received comprehensible information regarding the research methods and objectives, facilitated by the “Research information form for respondents”. Consent to participate was indicated by signing the “Consent form for participation in the study—Informed consent” after reviewing the information form. Participation was voluntary, with assurances of anonymity and the option to withdraw at any time during the study. The study received approval from the Ethics Committee of the Faculty of Medicine in Osijek on 16 September 2022 (CLASS: 602-04/21-08/02, REGISTRATION NUMBER: 2158-61-07-21-180).

### 2.2. Participants

**Figure 1:**
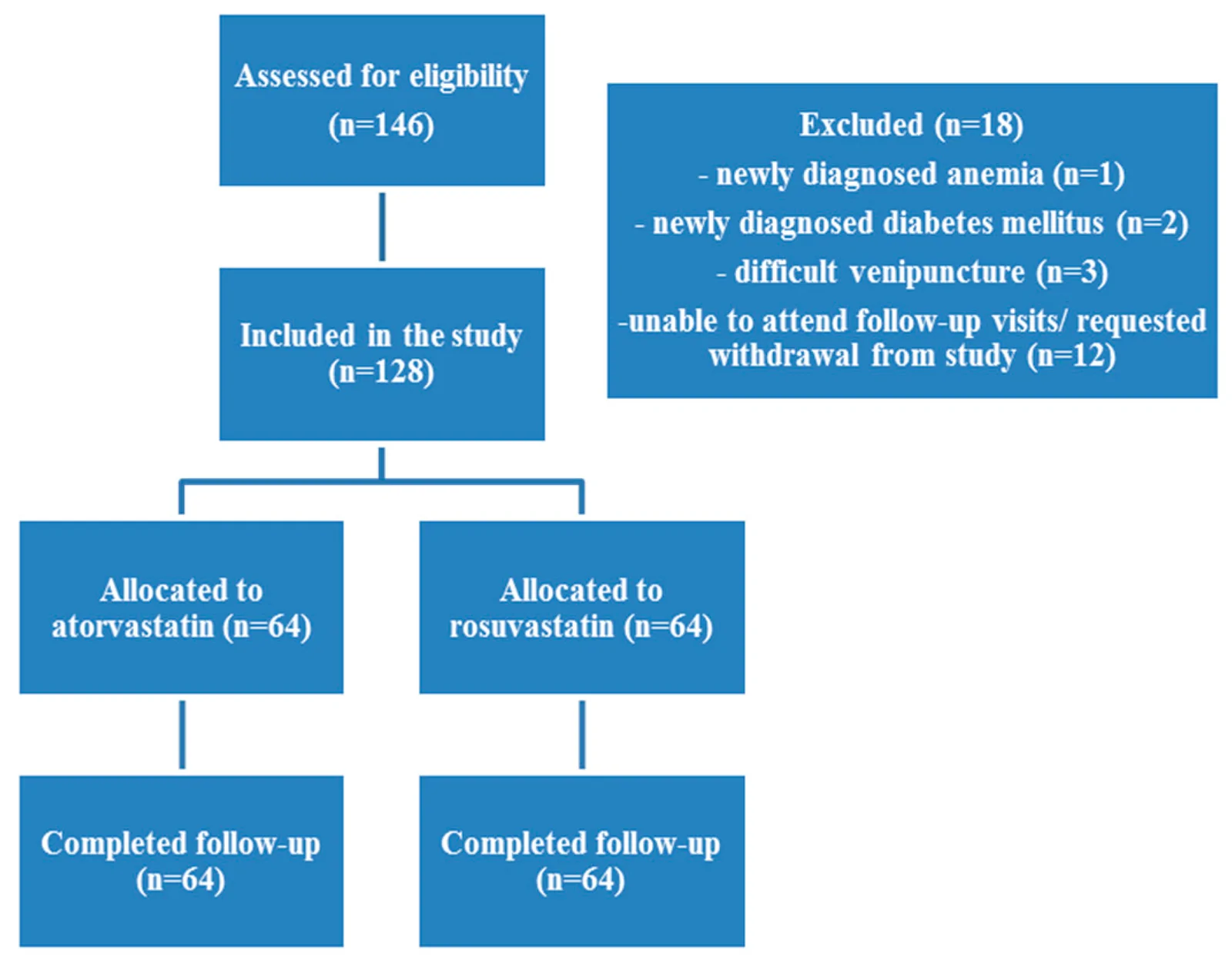
Flow chart of participant enrollment, allocation and follow-up.

A total of 146 individuals were assessed for eligibility; 18 of them were excluded, leaving 128 participants included in the final analysis. All study participants were of Caucasian ethnicity and permanent residents of the Osijek-Baranja County in Eastern Croatia. Participant enrollment, allocation and follow-up are shown in Figure 1.

The suitable participants for this study were postmenopausal women aged 45 to 70 years who had experienced amenorrhea for at least 12 months and had an indication for statin therapy in accordance with the 2019 ESC/EAS guidelines for dyslipidemia management and CVD prevention [31], the criteria of which remain fully endorsed by the 2025 focused update [36]. The exclusion criteria encompassed individuals younger than 45 years or older than 70 years, those with iatrogenic menopause, users of hormone replacement therapy, and individuals with uncontrolled arterial hypertension necessitating intensive therapeutic correction at the commencement of the study. Additional exclusion criteria were diabetes mellitus, rheumatological and immunological disorders, a history of cardiovascular incidents (acute myocardial infarction, cerebrovascular stroke, or transient ischemic attack), chronic kidney disease, active liver disease with elevated transaminase levels, dialysis requirements, alcoholism, malignant diseases, acute infections, sepsis, dementia, and neurological or psychiatric disorders necessitating antiepileptic, antidepressant, or antipsychotic medications. Furthermore, individuals using corticosteroid therapy, beta-blockers, levothyroxine, vitamin D, statin therapy, or therapies involving fenofibrate, ezetimibe, or proprotein convertase subtilisin/kexin type 9 inhibitors (PCSK9i) were considered unsuitable for inclusion in the study. Therapies that potentially increase the risk of myopathy and rhabdomyolysis due to statin use, such as itraconazole, ketoconazole, erythromycin, clarithromycin, amiodarone, verapamil, and diltiazem, as well as a personal history of allergy to statin therapy, were also exclusion criteria.

Following the initial measurement at the family medicine clinic, cardiovascular risk was evaluated using the Systematic Coronary Risk Estimation (SCORE2) table, and target LDL-C levels were established for each participant. Subsequently, statin therapy was initiated to treat dyslipidemia. Specifically, moderate-intensity statin therapy (atorvastatin 10 mg or rosuvastatin 5 mg) was prescribed if a reduction in LDL-C levels of 30–50% from baseline was required. Alternatively, high-intensity statin therapy (atorvastatin 40 mg or rosuvastatin 20 mg) was administered if a reduction of 50% or more from the baseline was necessary.

Further measurements were performed every 4 weeks, and the participants were monitored until the target LDL-C values were achieved or up to four follow-up measurements (total maximum duration of follow-up: 16 weeks) if the target LDL-C values were not achieved. To ensure compliance with the prescribed statin therapy, telephone checks/reminders were conducted every 7–10 days between follow-ups and were used to record side effects. Treatment adherence was also assessed at each visit by direct patient interview and pill count. Adherence ≥80% of prescribed doses was considered acceptable and required for inclusion in the final analysis. Regarding safety and tolerability, statin therapy was overall well tolerated in this cohort. Mild, transient adverse effects were reported by only 3 participants (2.3%), consisting exclusively of mild gastrointestinal symptoms: nausea was reported by 2 participants (1 in the atorvastatin group, 1 in the rosuvastatin group), and abdominal bloating was reported by 1 participant (atorvastatin group). No cases of myalgia, muscle weakness, or significant laboratory abnormalities (creatine kinase > 5 × ULN or transaminase levels > 3 × ULN) were recorded. No statin dose reductions or treatment discontinuations were required for any participant during the study period, and no serious adverse events occurred. At each measurement point, if the target LDL-C values were not achieved, the statin dose was increased (to the maximum recommended level, specifically up to 40 mg for rosuvastatin and 80 mg for atorvastatin, or to the maximum tolerated dose).

### 2.3. Procedures and Measurements

Data on fundamental characteristics were gathered via a survey specifically designed for this study and through a review of the electronic medical records and documentation available to the family doctor at the baseline visit. These data included age, employment status, education, smoking status, alcohol consumption, physical activity, age of menopause onset, duration of postmenopause period at the time of the study, presence of chronic diseases, use of chronic therapy, and allergies. Anthropometric measurements were taken: body mass (kg), body height (cm), and waist and hip circumferences (cm). The waist-to-hip ratio and body mass index (BMI) were calculated for each participant. Arterial blood pressure was measured using an Omron model M4 Connect AFib blood pressure monitor, which is recommended by the Croatian Society of Hypertension as a clinically validated blood pressure monitor.

Laboratory analyses were performed at baseline and during every monthly visit, including fasting venous blood sampling for complete blood count (CBC) with differential, creatine kinase (CK), glucose, urate, urea, creatinine, aspartate aminotransferase (AST), alanine aminotransferase (ALT), gamma-glutamyl transferase (GGT), TC, LDL-C, HDL-C, and triglicerydes (TG). Prior to blood sampling, participants received both written and verbal instructions to adhere to the following protocol: avoid strenuous physical activity for 48 h prior to sampling; maintain standard, non-greasy dietary habits for 24 h prior; fast, refrain from alcohol and coffee, and abstain from smoking for 10 h prior to blood collection. Venous blood samples were collected in the morning between 7:00 AM and 8:00 AM via venipuncture at the Medical-Biochemical Laboratory of the Health Center of Osijek-Baranja County (Croatia).

For the determination of urea, creatinine, glucose, urate, TC, HDL-C, LDL-C, TG, AST, ALT, GGT, and CK, blood samples were collected using 3 mL BD Vacutainer^®^ Clot Activator Tubes (CAT) (Franklin Lakes, NJ, USA). Following clot formation, samples were centrifuged for 10 min at 3000 rpm, and the separated serum was analyzed immediately. CBC with differential was evaluated from blood collected in 3 mL BD Vacutainer^®^ K2EDTA Tubes. CBC with differential was determined using a hematology analyzer DxH900 (Beckman Coulter, Indianapolis, IN, USA). The concentrations of glucose, urea, creatinine, urate, TC, TG, CK, AST, ALT, and GGT were determined using a photometric method on a DxC 700 AU analyzer (Beckman Coulter, Indianapolis, IN, USA). On the same analyzer, the concentration of HDL-C was determined using the homogeneous enzyme immunoinhibition method, and the method for determining the concentration of LDL-C depended on the concentration of TG. If the concentration of TG was ≤4.0 mmol/L, the concentration of LDL-C was calculated from the data on the concentration of TC, HDL-C, and TG according to the following formula: LDL-C = TC − (TG/2.2) − HDL-C. If the concentration of TG was >4.0 mmol/L, the concentration of LDL-C was determined directly using an enzymatic colorimetric test.

Venous blood samples collected using BD Vacutainer K2EDTA Tubes (6 mL) were used to determine the plasma levels of endocan and endoglin. After sampling, the samples were centrifuged for 10 min (3000 rpm), and a plasma sample was separated into two Eppendorf tubes, which were frozen within an hour of sampling, according to the manufacturer’s recommendations, at a minimum of −20 °C, and then stored in the Medical-Biochemical Laboratory of the Osijek-Baranja County Health Center (Croatia) until analysis. Commercial ELISA kits were used according to the manufacturer’s instructions, namely Human ESM1 ELISA Kit–Endocan (sensitivity: 10 pg/mL, range: 31.2–2000 pg/mL) and Human CD105 ELISA Kit–Endoglin (sensitivity: 10 pg/mL, range: 12.35–3000 pg/mL). Prior to use, all reagents were equilibrated to room temperature (18–25 °C). Both ELISA assays utilized a standard sandwich enzyme-linked immunosorbent technique. Samples and standards were incubated in 96-well plates pre-coated with target-specific monoclonal antibodies (anti-ESM1 or anti-CD105). Following washing, target proteins were detected using specific biotinylated antibodies, Avidin-Biotin-Peroxidase/Horseradish peroxidase–streptavidin conjugates and 3, 3′, 5, 5′-Tetramethylbenzidine substrate solution. The colorimetric reaction was stopped with an acidic solution, and absorbance was measured at 450 nm using a microplate reader within 30 min.

All laboratory parameters were assessed at each study point, with the exception of CK and plasma concentrations of endocan and endoglin. These specific parameters were measured prior to the initiation of statin therapy and subsequently after reaching the target LDL-C levels or at the conclusion of the 16-week follow-up period, if targets were not met.

### 2.4. Outcomes

The primary outcome of this study was the change in plasma concentrations of endocan and endoglin after statin therapy. Secondary outcomes included evaluating differences in biomarker response between atorvastatin and rosuvastatin, assessing the impact of achieving target LDL-C levels, and determining whether the change in LDL-C was independently associated with the magnitude of reduction in each biomarker. ROC curve analysis was performed as an exploratory outcome to evaluate the diagnostic and discriminatory performance of baseline, post-treatment, and absolute change values of endocan and endoglin in identifying non-achievers of target LDL-C levels.

### 2.5. Statistical Analyses

Sample size estimation was performed prior to study initiation using G*Power software (version 3.1.2). To detect a medium effect size for repeated measures of numerical variables within each treatment group (atorvastatin and rosuvastatin arms across up to four measurement time points), with a significance level α = 0.05 and a statistical power (1 -β) of 0.95, a minimum sample size of 36 participants per group was required. Furthermore, to detect a medium effect size for differences in numerical variables between the two independent treatment groups (atorvastatin vs. rosuvastatin) with α = 0.05 and a statistical power of 0.80, a minimum sample size of 64 participants per group (total N = 128) was necessary. Therefore, the total target sample size for the study was set to a minimum of 128 participants.

Categorical variables were presented as absolute and relative frequencies. The normality of the distribution of continuous variables was assessed using the Shapiro–Wilk test. Due to the non-normal distribution, these variables were expressed as medians and interquartile ranges. Mann–Whitney U test was used for comparison of continuous variables and the Chi-square test for comparison of categorical variables. The Hodges–Lehmann estimate was used to determine the median difference with the corresponding 95% confidence intervals. The non-parametric Wilcoxon signed-rank test was used to examine intra-group changes over time. Spearman’s rank correlation coefficient was used to determine the direction and strength of the association between the absolute changes in plasma endothelial biomarkers and changes in lipid profile indicators. Multivariate linear regression analysis was performed to determine whether changes in LDL-C concentrations were independently associated with changes in endocan and endoglin levels, adjusting for baseline biomarker concentrations, baseline LDL-C, statin type and dose, age, BMI, smoking status, and treatment duration. ROC curve analysis was conducted to assess the potential discriminatory ability of plasma endocan and endoglin concentrations in identifying postmenopausal women who failed to achieve the recommended target LDL-C values. A *p*-value < 0.05 was considered statistically significant, and all *p*-values were two-sided.

## 3. Results

### 3.1. Demographic, Anthropometric and Laboratory Characteristics of Participants

The median age of the participants was 60 years (IQR 56–64 years). Menopause occurred before the age of 50 years in 43% of the participants, whereas 57% experienced menopause at or after 50 years of age. Among the entire sample, 45% of the participants were non-smokers, and in the past year, 54% of the participants reported alcohol consumption, with the most common frequency being several times annually (35%), and daily consumption being rare (4%).

The participants had a median body mass of 73 kg (IQR 66–82 kg), and the median BMI was 27.02 kg/m^2^ (IQR 23.94–30.85 kg/m^2^). Participants engaged in moderate physical activity for a median of 2 days per week, while sedentary behavior ranged from 60 to 90 min daily. Table 1 provides detailed information on the participants’ demographics, anthropometrics, lifestyle, and activity levels.

The baseline demographic and anthropometric characteristics were comparable between the treatment groups (atorvastatin vs. rosuvastatin). Analysis of laboratory parameters before the introduction of statin therapy showed that the values of GGT (Mann–Whitney U test, *p* = 0.009), TC (Mann–Whitney U test, *p* = 0.003), and LDL-C (Mann–Whitney U test, *p* = 0.01) were significantly higher in the group of participants receiving rosuvastatin, while the values of TG were significantly higher in the group of participants receiving atorvastatin (Mann–Whitney U test, *p* < 0.001). At the end of the study, no statistically significant differences were observed in repeated measurements of laboratory parameters with respect to the used statin therapy.

### 3.2. Cardiovascular Risk, Achievement of LDL-C Target Values and Non-HDL-C Values in Participants

In 78% of the study participants, cardiovascular risk was classified as high or very high according to the SCORE2 algorithm table. No statistically significant differences in cardiovascular risk were identified among the participants concerning the type of administered statin therapy (Table 2).

The study found a statistically significant difference in non-HDL cholesterol (non-HDL-C) and LDL-C values between the treatment groups before the introduction of statin therapy (Mann–Whitney U test, non-HDL-C *p* = 0.001; LDL-C *p* = 0.01), with higher non-HDL-C and LDL-C values recorded in the group receiving rosuvastatin. After achieving the target LDL-C values or at the end of follow-up (16 weeks), no significant differences were found between the treatment groups in non-HDL-C and LDL-C values. No significant differences were observed between the treatment groups in terms of estimated cardiovascular risk, expressed as a percentage according to the SCORE2 algorithm, or target LDL-C values. The median treatment duration before the final biomarker measurement was 12 (IQR 8–12) weeks. Final biomarker measurements were performed at 4 weeks in 4 (3.1%) participants, at 8 weeks in 39 (30.5%), at 12 weeks in 62 (48.4%), and at 16 weeks in 23 (18.0%). Treatment duration did not significantly differ between the atorvastatin and rosuvastatin groups (Table 3).

In both groups, LDL-C decreased after using statin therapy, and target LDL-C values were achieved in a total of 55.5% of the participants with no significant differences according to the type of statin therapy used (Table 4).

In both treatment groups, a significant decrease in non-HDL-C levels was observed during follow-up (Wilcoxon test, *p* < 0.001 for both groups) (Table 5).

### 3.3. The Influence of Statin Therapy on the Concentration of Plasma Markers of Endothelial Dysfunction—Endocan and Endoglin

The study did not found differences in plasma concentrations of endocan and endoglin based on the type of statin therapy administered, both before and after the initiation of statin treatment (Table 6).

After reaching the LDL-C target values or at the end of the follow-up period, if the LDL-C target values were not reached, statistically significant decreases in the plasma concentrations of the endothelial dysfunction markers endocan and endoglin were recorded in both the atorvastatin (Wilcoxon test, *p* < 0.001) and rosuvastatin (Wilcoxon test, *p* < 0.001) groups (Table 7). No significant differences in the changes (Δ values) in the observed indicators were found between the test groups treated with atorvastatin and rosuvastatin (Table 8).

Treatment duration was significantly shorter in participants who achieved the LDL-C goal than in those who did not (Mann–Whitney U test, *p* < 0.001). No significant differences were found in serum endocan and endoglin concentrations, either before statin treatment or at the final measurement, between participants who achieved and those who did not achieve the LDL-C goal (Table 9).

### 3.4. Correlation Between Changes in Markers of Endothelial Dysfunction (Endocan and Endoglin) and Changes in Lipid Indicators

In the total sample, no significant associations were found between changes in endothelial biomarkers and changes in lipid parameters. In the group of participants who did not reach the target LDL-C value, the change in endoglin was significantly but weakly negatively associated with the change in LDL-C (ρ = −0.252). In the group of participants who achieved the target LDL-C value, significant and positive associations were observed between the change in endocan concentration and the change in LDL-C (ρ = 0.337) and TG (ρ = 0.326) levels. The change in endoglin levels was significantly and positively correlated with changes in TC (ρ = 0.277) and TG (ρ = 0.340) levels. The established significant correlations were mostly weak (ρ < 0.40) (Table 10).

Detailed data on initial and final statin doses, treatment intensity, and dose escalation are provided in Supplementary Table S1, while statin dose distributions across consecutive 4-week treatment intervals are presented in Supplementary Table S2. At treatment initiation, high-intensity therapy was used in 67.2% of participants receiving atorvastatin and 71.9% receiving rosuvastatin, increasing to 73.4% and 78.1%, respectively, at the final measurement. Dose escalation was required in 71.9% of participants receiving atorvastatin and 79.7% receiving rosuvastatin.

In multivariate linear regression analysis, change in LDL-C was not independently associated with change in endocan concentration (B = −51.24, 95% CI −342.30 to 239.81; *p* = 0.73). In contrast, change in LDL-C was significantly associated with change in endoglin concentration (B = 175.05, 95% CI 31.00 to 319.10; *p* = 0.018), with greater reductions in LDL-C being associated with greater reductions in endoglin. Both models were adjusted for baseline concentration of the respective biomarker, baseline LDL-C, type of statin, final statin dose, age, BMI, current smoking, and duration of treatment. The baseline concentration of the corresponding biomarker was significantly associated with its change in both models (*p* < 0.001), while the other included covariates were not statistically significant (Table 11).

After adjustment for the corresponding baseline biomarker concentration, baseline LDL-C, triglycerides, and GGT, the randomized statin treatment group was not significantly associated with final endocan (B = −185.78, 95% CI −479.57 to 108.01; *p* = 0.21) or final endoglin concentrations (B = 100.03, 95% CI −55.18 to 255.44; *p* = 0.20). Baseline concentration of the corresponding biomarker was significantly associated with its final concentration in both models (*p* < 0.001) (Supplementary Table S3).

ROC analysis was used to assess the discriminatory ability of plasma concentrations of endocan and endoglin to identify patients who did not achieve the target LDL-C value. Endocan and endoglin, regardless of the time of determination (before therapy, after therapy or as a change during follow-up), did not show significant discriminatory ability (Table 12).

## 4. Discussion

This study examined how the concentration of plasma markers of endothelial dysfunction, endocan and endoglin, is affected by the use of atorvastatin and rosuvastatin in primary prevention in postmenopausal women, and whether there is a difference depending on the type of statin therapy used. Furthermore, it investigated whether reaching target LDL-C values was a prerequisite for the decrease in endocan and endoglin plasma concentrations. Additionally, the potential relationship between variations in the subjects’ lipid profiles and changes in the plasma amounts of endocan and endoglin was examined. Finally, this study aimed to determine whether the magnitude of change in LDL-C concentration is independently associated with changes in endocan and endoglin levels after adjusting for baseline biomarker levels, baseline LDL-C, statin type and dose, treatment duration, and demographic and clinical covariates.

The majority of participants were categorized as having a high or very high cardiovascular risk after their cardiovascular risk was assessed utilizing the SCORE2 algorithm. The increase in cardiovascular risk in postmenopausal women has been confirmed by numerous studies, and the accelerated development of ASCVD in postmenopausal women is considered to be based on hormonal, metabolic, vascular, and many other changes [37]. The median age of menopause onset in our participants was 50 years, which corresponds to the average age of menopause reported in many studies conducted to date. The median age at menopause among white women in Europe is between 50 and 52 years. These ages appear to differ based on race and ethnicity and are impacted by genetic, demographic, and lifestyle factors, such as dietary habits and alcohol consumption [38].

Looking at all demographic and anthropometric parameters, data on smoking status, alcohol consumption, and physical activity, as well as estimated cardiovascular risk and LDL-C target values, there were no differences between the groups (atorvastatin vs. rosuvastatin) at inclusion in the study, which means that the comparability of these groups at the beginning of the study was adequate. Given that the median duration of menopause in our subjects was 10 years (IQR 5–14), they can be classified as being in a stable postmenopausal state rather than the perimenopausal phase, during which the hormonal fluctuations that most acutely affect the vascular endothelium are prominent [39].

In both treatment groups, LDL-C and non-HDL-C concentrations decreased after using statin therapy, and LDL-C target values were achieved in 55.5% of the subjects, with no significant difference observed between the observed therapeutic groups. This confirms the known fact that atorvastatin and rosuvastatin are effective in reducing LDL-C concentrations, and with the additional use of drugs such as ezetimibe, the LDL-C target value can be achieved in an even greater number of people. Studies such as the LODESTAR Trial, including comparative meta-analyses and direct comparisons, have demonstrated that rosuvastatin frequently achieves a marginally greater percentage decrease in LDL-C levels than atorvastatin, even when administered at the same or lower milligram dosages [40,41]. In our study, rosuvastatin consumption resulted in a greater reduction in LDL-C concentration, but no significant differences were observed in LDL-C concentrations at the end of the follow-up period.

While current dyslipidemia treatment guidelines specify target LDL-C levels for various cardiovascular risk categories, many studies have highlighted the significant role of triglyceride and non-HDL-C levels in estimating a residual cardiovascular risk. Patients in key statin trials still had a significant residual cardiovascular risk even when reducing LDL-C decreased the relative risk of ASCVD. Elevated ApoB 100 levels, which directly reflect the total amount of atherogenic particles regardless of size and density, are correlated with this higher risk. Because of this, ApoB is a more reliable indicator of the risk of ASCVD than conventional lipid tests, which do not take particle size or quantity into consideration. The AMORIS study demonstrated that ApoB levels linked better with the severity of coronary artery disease (CAD) than LDL-C levels and were a greater predictor of ASCVD than LDL-C, independent of LDL-C [42].

Despite substantial evidence supporting the superiority of ApoB as an ASCVD marker, its routine use in clinical practice, particularly for primary prevention, remains limited because it is not included in standard lipidogram measurements used in primary clinical practice. Since research indicates that non-HDL-C values, which include cholesterol carried by LDL, triglyceride-rich lipoproteins (TRL), and lipoprotein (a), are superior to LDL-C alone in predicting major adverse cardiovascular events (MACE) in patients treated with statins for ASCVD or those at high risk of ASCVD, it is advised to use non-HDL-C values for the aforementioned purposes [43]. In our study, both treatment groups showed a significant reduction in non-HDL-C levels by the end of the study (Wilcoxon test, *p* < 0.001 for both groups) with no difference observed between the treatment groups.

Plasma endocan levels increase with age, and in women, these levels further increase with the onset of menopause [44]. In individuals with arterial hypertension, chronic kidney disease, diabetes, and coronary heart disease, plasma endocan levels are also higher, along with increased levels of adhesion molecules such as ICAM-1 and VCAM-1, a phenomenon that also occurs during the postmenopausal phase, even in the absence of chronic illnesses. The decline in estrogen levels during the postmenopausal period results in endothelial dysfunction, leading to a marked increase in circulating ICAM-1 and VCAM-1 levels. These elevated adhesion molecules contribute to systemic inflammation and leukocyte recruitment, thereby increasing cardiovascular and metabolic risks [22]. Earlier studies have indicated that in postmenopausal women, the rise in plasma endocan levels is further driven by metabolic syndrome, and there is a strong correlation between plasma endocan levels and carotid artery intima-media thickness (CIMT) [45]. Evidence also suggests a link between plasma endocan levels and increased BMI and a higher percentage of visceral fat, which is frequently observed in postmenopausal women [22]. The simultaneous presence of high plasma endocan levels and elevated TC and LDL-C levels has been identified as a predictor of CAD development and reduced coronary flow [46].

Studies indicate that circulating endoglin levels are also elevated during aging. In postmenopausal women, the depletion of estrogen removes its protective upregulatory effect on membrane-bound endoglin, which is accompanied by an increase in the enzymes (metalloproteinases) that cleave endoglin, releasing it into the bloodstream. Elevated plasma endoglin acts as an antagonist to membrane-bound endoglin by trapping growth factors, disrupting crucial vascular (TGF-β1 and eNOS) signaling pathways, and promoting vasoconstriction. Because of these mechanisms, high plasma endoglin levels are often considered to be an indicator of vascular dysfunction and CVD development in aging women [47,48,49]. A meta-analysis conducted to investigate the association between endoglin and certain cardiovascular and metabolic disorders showed that plasma endoglin concentrations are elevated and may reflect endothelial damage, particularly in patients with diabetes and heart failure [50,51].

Our study showed that statin therapy decreased plasma endocan (Wilcoxon test, *p* < 0.001) and endoglin (Wilcoxon test, *p* < 0.001) levels in both treatment groups. Although the rosuvastatin group had higher baseline TC and LDL-C levels and a larger absolute non-HDL reduction (rosuvastatin: −2.6 vs. atorvastatin: −1.9), the changes (Δ values) in endocan and endoglin did not differ significantly between treatment arms (rosuvastatin vs. atorvastatin). No statistically significant difference was observed between the two statins regarding their effect on lowering these endothelial biomarkers. Furthermore, decreased plasma endocan and endoglin levels occurred independently of participants reaching target LDL-C levels. While this finding suggests that reductions in these biomarkers may not be strictly tied to degree of lipid lowering, it cannot serve as definitive proof of direct, lipid-independent pleiotropic effects, particularly in the absence of an untreated control group or direct functional vascular assessments.

To further clarify the mechanisms driving the observed vascular changes, multivariate linear regression models were employed to evaluate the independent relationship between LDL-C reduction and changes in biomarker concentrations. Our results demonstrate a clear divergence between endocan and endoglin dynamics in response to statin therapy. The reduction in endocan concentration was not independently associated with the magnitude of LDL-C lowering (B = −51.24, *p* = 0.730), even after adjusting for baseline biomarker levels, baseline LDL-C, statin type and dose, age, BMI, smoking status, and treatment duration. This finding strongly supports the hypothesis that statin-induced modulation of endocan is predominantly mediated by lipid-independent pathways. In contrast, the change in endoglin levels was significantly and positively associated with the magnitude of LDL-C reduction (B = 175.05, 95% CI 31.00 to 319.10, *p* = 0.018). Patients who achieved greater reductions in LDL-C experienced significantly larger decreases in circulating endoglin. Notably, in both regression models, the baseline concentration of the respective biomarker was the only other significant predictor of its overall change. This strong association indicates that women presenting with higher baseline levels of endothelial activation and dysfunction derive a proportionally greater biomarker response from statin initiation, regardless of statin type (atorvastatin vs. rosuvastatin) or final dose.

In addition to inhibiting HMG-CoA reductase to lower cholesterol production, statins provide direct protective benefits to the vascular endothelium. These pleiotropic effects include the upregulation of eNOS, reducing oxidative stress, stabilization of atherosclerotic plaques, and inhibition of the thrombogenic response [52,53]. In postmenopausal women, where declining estrogen affects NO pathways, these non-lipid-modifying statin properties have been hypothesized to help maintain vascular balance [54]. The observed reduction in endocan and endoglin concentrations in our study aligns with previous in vitro and in vivo reports, which also documented lower biomarker levels following statin administration, particularly in patients with acute myocardial infarction [55,56].

In line with existing understanding, ROC analysis revealed that plasma endocan and endoglin concentrations are not useful clinical predictors for identifying patients who will not reach target LDL-C values. Namely, endocan and endoglin are markers of vascular endothelial stress, for which lipid molecules are not solely responsible [45,47]. Furthermore, LDL-C reduction is influenced by various factors, including genetic variations, statin dosage, hepatic and renal pharmacokinetics, bioavailability, interactions with other medications, and adherence to therapy, making the link between lipid lowering and biomarker modulation complex and non-linear [52,53,57].

### Study Limitations

This study has several notable strengths. It focused on a well-defined cohort of postmenopausal women, all adhering to consistent inclusion criteria, with lipid levels regularly assessed at each appointment. Furthermore, it addresses a vital gap in the current research by focusing on a particularly susceptible group of postmenopausal women, in whom hormonal decline combined with metabolic changes increases cardiovascular risk. The study’s structure featured two comparable treatment groups allowing for a reliable direct comparison of their effects on the vascular endothelium.

However, we acknowledge several limitations of the study. First, the follow-up duration was limited to 16 weeks. Although this period was sufficient to observe significant reductions in lipid parameters and endothelial biomarkers, extending the follow-up period would be beneficial to determine whether the observed decreases in endocan and endoglin levels are sustained over time and how they affect long-term major adverse cardiovascular events (MACE). Second, the study lacked a non-treated control group of postmenopausal women, which restricts the ability to completely rule out potential confounding variations in biomarker levels. Nonetheless, the comparative design between the two well-matched statin groups partially mitigated this issue. Future prospective studies incorporating an untreated control cohort are warranted to further isolate statin-specific mechanisms from potential background physiological changes. Additionally, while non-HDL-C was used as an indicator of atherogenic particles, ApoB was not measured. Future research incorporating ApoB could provide a more precise assessment of the total atherogenic particle burden and help clarify its direct relationship with changes in endothelial biomarkers, such as endocan and endoglin.

## 5. Conclusions

The use of atorvastatin and rosuvastatin for the primary prevention of cardiovascular events in postmenopausal women significantly lowered plasma levels of endocan and endoglin, with no statistically significant differences observed between the two statin regimens. Statin therapy leads to a reduction in plasma endocan and endoglin concentrations in postmenopausal women, with the decrease in endoglin being directly associated with the degree of LDL-C reduction, whereas the decline in endocan occurs independently of changes in lipid levels. The magnitude of reduction for both biomarkers was highest in women with higher baseline concentrations, regardless of the type or dose of statin used. Although plasma endocan and endoglin concentrations were not predictive of achieving target LDL-C levels, their marked reduction highlights a favorable response to statin therapy in the early primary prevention of CVD in postmenopausal women.

## Figures and Tables

**Table 1:** Demographic, anthropometric and lifestyle characteristics of participants.

Variable	Median (IQR) or N (%) (*n* = 128)
Age (years)	60 (56–64)
Age at menopause onset (years)	50 (48–52)
Duration of menopause (years)	10 (5–14)
Body mass (kg)	73 (66–82)
Body mass index (kg/m^2^)	27.02 (23.94–30.85)
Waist circumference (cm)	88 (80–97)
Hip circumference (cm)	106 (101–112)
Waist-to-hip ratio	0.83 (0.77–0.88)
Systolic blood pressure (mmHg)	126 (116–140)
Diastolic blood pressure (mmHg)	84 (76–90)
Heart rate (beats per min)	75 (70–80)
Smoking status	
Non-smoker	58 (45)
Regular smoker	41 (32)
Occasional smoker	6 (5)
Former smoker	23 (18)
Alcohol consumption (past 12 months)	
None	59 (46)
Daily	5 (4)
Weekly	9 (7)
Monthly	10 (8)
Several times annually	45 (35)
Employment status	
Employed	74 (58)
Unemployed	6 (5)
Retired	48 (38)
Education level	
Primary school	4 (3)
High school	85 (66)
Associate degree	12 (9)
University degree	23 (18)
Professional specialist	2 (2)
Doctorate degree	2 (2)

Categorical variables are presented as absolute and relative frequencies; continuous variables are presented as median (interquartile range).

**Table 2:** Cardiovascular risk distribution among study participants according to statin therapy group.

	N (%)			*p* *
	Atorvastatin	Rosuvastatin	Total	
Cardiovascular risk				
Moderate	16 (25)	16 (25)	32 (25)	>0.99
High	24 (38)	24 (38)	48 (38)	
Very high	24 (38)	24 (38)	48 (38)	

* χ^2^ test.

**Table 3:** Treatment duration and differences in baseline and final LDL-C, non-HDL-C, and target LDL-C values between atorvastatin and rosuvastatin treatment groups.

	Median (IQR)		^†^ Difference	95% Confidence Interval	*p* *
	Atorvastatin (n = 64)	Rosuvastatin (n = 64)			
Treatment duration (weeks)	12 (8–12)	12 (8–12)	0	0 to 0	0.27
Baseline LDL-C value (mmol/L)	4.1 (3.6–4.6)	4.6 (3.8–5.2)	0.4	0.1 to 0.7	**0.01**
Final LDL-C value (mmol/L)	2.0 (1.7–2.4)	1.9 (1.7–2.3)	−0.1	−0.2 to 0.1	0.41
Baseline non-HDL-C value (mmol/L)	4.7 (4.2–5.3)	5.3 (4.6–6.0)	0.5	0.2 to 0.9	**0.001**
Final non-HDL-C value (mmol/L)	2.8 (2.4–3.2)	2.6 (2.4–2.9)	−0.1	−0.3 to 0.1	0.30
Target LDL-C value (mmol/L)	1.8 (1.4–2.2)	1.8 (1.4–2.2)	0	0 to 0	>0.99

* Mann–Whitney U test; ^†^ Hodges–Lehmann estimate of median difference. Bold value is statistically significant.

**Table 4:** Distribution of participants according to achieved target values and type of statin therapy.

		N (%)			*p* *
		Atorvastatin	Rosuvastatin	Total	
LDL-C target value					
Yes	37 (57.8)		34 (53.1)	71 (55.5)	0.59
No	27 (42.2)		30 (46.9)	57 (44.5)	

* χ^2^ test.

**Table 5:** Changes in non-HDL cholesterol values before treatment and after reaching LDL-C target values or at the end of follow-up period by treatment group.

	Median (IQR)		^†^ Difference	95% Confidence Interval	*p* *
	Before Statin Treatment	After Reaching LDL-C Target Values or at the End of Follow-Up			
Atorvastatin					
non-HDL cholesterol (mmol/L)	4.7 (4.2–5.3)	2.8 (2.4–3.2)	−1.9	−2.1 to −1.6	**<0.001**
Rosuvastatin					
non-HDL cholesterol (mmol/L)	5.3 (4.6–6.0)	2.6 (2.4–2.9)	−2.6	−2.8 to −2.3	**<0.001**

* Wilcoxon test; ^†^ Hodges–Lehmann estimate of median difference. Bold value is statistically significant.

**Table 6:** Differences in plasma concentrations of endocan and endoglin between therapy groups before and after treatment.

	Median (IQR)		^†^ Difference	95% Confidence Interval	*p* *
	Atorvastatin (*n* = 64)	Rosuvastatin (*n* = 64)			
Before statin treatment					
Endocan (pg/mL)	1045.3 (524.4–1706.3)	1349.6 (599.1–1723.6)	110	−137.3 to 407.9	0.36
Endoglin (pg/mL)	1346.9 (1047.8–1722.5)	1510.6 (1227.4–1979.2)	192.6	−5.1 to 403.2	0.05
After reaching LDL-C target values or at the end of follow-up period					
Endocan (pg/mL)	405.0 (273.9–743.2)	384.9 (300.9–853.6)	28.6	−52.2 to 97.1	0.48
Endoglin (pg/mL)	919.1 (601.7–1253.6)	980.8 (779.5–1415.4)	97.9	−68 to 263	0.23

* Mann–Whitney U test; ^†^ Hodges–Lehmann estimate of median difference.

**Table 7:** Changes in the plasma concentration of endocan and endoglin before the introduction of statin therapy and after reaching the target values of LDL cholesterol or at the end of the follow-up within the treatment groups.

	Median (IQR)		^†^ Difference	95% Confidence Interval	*p* *
	Before Statin Treatment	After Reaching LDL-C Target Values or at the End of the Follow-Up			
Atorvastatin					
Endocan (pg/mL)	1045.3 (524.4–1706.3)	405.0 (273.9–743.2)	−557	−761.1 to −384.8	**<0.001**
Endoglin (pg/mL)	1346.9 (1047.8–1722.5)	919.1 (601.7–1253.6)	−398.9	−489.2 to −298.6	**<0.001**
Rosuvastatin					
Endocan (pg/mL)	1349.6 (599.1–1723.6)	384.9 (300.9–853.6)	−634.2	−856.3 to −456.2	**<0.001**
Endoglin (pg/mL)	1510.6 (1227.4–1979.2)	980.8 (779.5–1415.4)	−466.2	−606.4 to −349.9	**<0.001**

* Wilcoxon test; ^†^ Hodges–Lehmann estimate of median difference. Bold value is statistically significant.

**Table 8:** Differences in changes in plasma concentrations of endocan and endoglin (Δ values) between the atorvastatin and rosuvastatin groups.

	Median (IQR)		^†^ Difference	95% Confidence Interval	*p* *
	Atorvastatin (*n* = 64)	Rosuvastatin (*n* = 64)			
ΔEndocan	−413.8 (−1015.9 to −135.3)	−585.5 (−1104.3 to −134.2)	−34.3	−269.8 to 145.1	0.68
ΔEndoglin	−331.1 (−741.4 to −104.0)	−396.3 (−837.6 to −169.3)	−72.3	−206.9 to 66.6	0.29

* Mann–Whitney U test; ^†^ Hodges–Lehmann estimate of median difference.

**Table 9:** Differences in treatment duration and endocan and endoglin concentrations according to LDL-C target attainment.

	Median (IQR)		^†^ Difference	95% Confidence Interval	*p* *
	LDL-C Target Values—Not Achieved	LDL-C Target Values—Achieved			
Treatment duration (weeks)	12 (12–12)	8 (8–12)	−4	−4 to −4	**<0.001**
Before statin treatment					
Endocan (pg/mL)	1099.9 (572.7–1632.3)	1343.3 (529.9–1755.9)	119.9	−126.9 to 405.3	0.36
Endoglin (pg/mL)	1401.9 (1237.6–1914.02)	1468.5 (1078.1–1782.6)	−45.2	−244.1 to 173.9	0.71
After reaching LDL-C target values or at the end of follow-up period					
Endocan (pg/mL)	386.5 (292.3–728.3)	426.4 (288.7–812.5)	31.7	−41.6 to 119.1	0.39
Endoglin (pg/mL)	935.9 (765.3–1380.6)	988.5 (648.3–1330.9)	−39.8	−212.1 to 132.2	0.66

* Mann–Whitney U test; ^†^ Hodges–Lehmann estimate of median difference. Bold value is statistically significant.

**Table 10:** Correlation of changes in endothelial biomarkers with changes in lipid indicators towards achieving LDL cholesterol target values.

	Spearman’s Rho Correlation Coefficient (*p* Values)	
	ΔEndocan	ΔEndoglin
All participants		
ΔLDL-C	0.159 (0.07)	−0.012 (0.89)
Δtotal cholesterol	0.102 (0.25)	0.060 (0.50)
Δtriglycerides	0.119 (0.18)	0.145 (0.10)
LDL-C target values—not achieved		
ΔLDL-C	−0.004 (0.97)	**−0.252 (0.03)**
Δtotal cholesterol	−0.038 (0.75)	−0.118 (0.33)
Δtriglycerides	−0.032 (0.79)	−0.024 (0.84)
LDL-C target values—achieved		
ΔLDL-C	**0.337 (0.01)**	0.240 (0.07)
Δtotal cholesterol	0.265 (0.05)	**0.277 (0.04)**
Δtriglycerides	**0.326 (0.01)**	**0.340 (0.01)**

Bold value is statistically significant.

**Table 11:** Multivariate linear regression analysis of factors associated with changes in serum endocan and endoglin concentrations.

Predictor	ΔEndocan B (95% CI)	*p* Value	ΔEndoglin B (95% CI)	*p* Value
ΔLDL-C	−51.24 (−342.30 to 239.81)	0.73	175.05 (30.998 to 319.10)	**0.018**
^†^ Baseline biomarker	−0.738 (−0.782 to −0.693)	**<0.001**	−0.748 (−0.829 to −0.668)	**<0.001**
Baseline LDL-C	−12.38 (−323.30 to 298.53)	0.94	30.51 (−122.36 to 183.38)	0.69
^‡^ Statin type	−222.49 (−651.43 to 206.45)	0.31	168.65 (−42.86 to 380.16)	0.12
Final statin dose	0.03 (−10.02 to 10.08)	0.99	2.12 (−2.87 to 7.12)	0.40
Age	−17.56 (−49.40 to 14.29)	0.28	−3.95 (−19.80 to 11.90)	0.62
BMI	6.52 (−23.22 to 36.25)	0.67	−1.76 (−16.42 to 12.90)	0.81
Treatment duration (weeks)	13.26 (−43.12 to 69.64)	0.64	23.36 (−4.54 to 51.26)	0.10
Current smoking	−55.55 (−386.64 to 275.55)	0.74	−22.42 (−185.55 to 140.72)	0.79
Model statistics	R^2^ = 0.904; adjusted R^2^ = 0.897; *p* < 0.001		R^2^= 0.752; adjusted R^2^ = 0.733; *p* < 0.001	

BMI = body mass index; CI = confidence interval; LDL-C = low-density lipoprotein cholesterol. Values are unstandardized regression coefficients (B) with 95% confidence intervals (CI). ^†^ Baseline biomarker refers to baseline endocan in the ΔEndocan model and baseline endoglin in the ΔEndoglin model. Δ represents the change from baseline to the end of follow-up (final − baseline). ^‡^ Statin type was coded as 1 = atorvastatin and 2 = rosuvastatin. Current smoking was coded as 0 = non-smoker/former smoker and 1 = occasional/regular smoker. Bold values indicate statistical significance. Bold value is statistically significant.

**Table 12:** ROC analysis of the assessment of the discriminating abilities of endocan and endoglin to identify participants who do not reach the target value of LDL cholesterol.

	AUC	95% CI	Sensitivity	Specificity	Cut-Off	Youden Index	*p* Value (AUC)
Endoglin—before therapy	0.519	0.429–0.608	78.9	35.2	>1200.1	0.142	0.71
Endoglin—after therapy	0.523	0.433–0.612	22.8	91.5	>1590.5	0.144	0.66
ΔEndoglin	0.537	0.447–0.626	52.6	59.2	>337.9	0.118	0.47
Endocan—before therapy	0.547	0.457–0.635	71.9	42.3	≤1507.1	0.142	0.36
Endocan—after therapy	0.544	0.454–0.632	73.7	40.8	≤615.8	0.145	0.39
ΔEndocan	0.512	0.423–0.602	40.4	69.0	>274.4	0.093	0.81

AUC—Area under the curve.

## Data Availability

The original contributions presented in this study are included in this article. Further inquiries can be directed to the corresponding author, T.B., upon reasonable request.

## Supplementary Materials

The following supporting information can be downloaded at: https://www.mdpi.com/article/10.3390/biomedicines14091921/s1; Table S1: Statin doses, treatment intensity, and dose escalation according to treatment group; Table S2: Distribution of statin doses across consecutive 4-week treatment intervals according to treatment group; Table S3: Adjusted linear regression analyses of final endocan and endoglin concentrations according to statin treatment.

## Abbreviations

The following abbreviations are used in this manuscript:

WHO	World Health Organisation
CVD	Cardiovascular disease
NO	Nitric oxide
HDL-C	High-density lipoprotein cholesterol
LDL-C	Low-density lipoprotein cholesterol
TC	Total cholesterol
PAI-1	Plasminogen activator inhibitor-1
ApoB	Apolipoprotein B
TNF-α	Tumor necrosis factor alpha
IL-6	Interleukin-6
IL-1β	Interleukin-1 beta
ICAM-1	Intercellular adhesion molecule 1
VCAM-1	Vascular cell adhesion molecule
eNOS	Endothelial nitric-oxide synthase
sEng	Soluble endoglin
ASCVD	Atherosclerotic cardiovascular disease
HMG-CoA	3-hydroxy-3-methylglutaryl-coenzyme A
PCSK9i	Proprotein convertase subtilisin/kexin type 9 inhibitors
SCORE2	Systematic Coronary Risk Estimation
BMI	Body mass index
CBC	Complete blood count
CK	Creatin kinase
AST	Aspartate aminotransferase
ALT	Alanine aminotransferase
GGT	Gamma-glutamyl transferase
TG	Triglycerides
Non-HDL-C	Non-HDL cholesterol
LODESTAR	Low-density Lipoprotein Cholesterol-Targeting Statin Therapy Versus Intensity-Based Statin Therapy in Patients With Coronary Artery Disease
CAD	Coronary artery disease
TRL	Triglyceride-rich lipoproteins
MACE	Major adverse cardiovascular events
CIMT	Carotid artery intima-media thickness
