# Supplementary material for: The Influence of Statin Therapy on Markers of Endothelial Dysfunction, Endocan and Endoglin, in Postmenopausal Women—A Prospective Study
Source: Biomedicines. 2026 Aug 27;14(9):1921. doi: 10.3390/biomedicines14091921 (PMC13604219; doi:10.3390/biomedicines14091921)
Supplement: Supplementary file 1 [file biomedicines-14-01921-s001.zip › biomedicines-4477454-supplementary.pdf]

Supplementary Table S1. Statin doses, treatment intensity, and dose escalation according to treatment group.

Treatment characteristics	Atorvastatin (n = 64)	Rosuvastatin (n = 64)
Initial statin dose, n (%)		
5 mg	-	18 (28.1)
10 mg	21 (32.8)	-
20 mg	-	45 (70.3)
30 mg	-	1 (1.6)
40 mg	43 (67.2)	-
Initial treatment intensity, n (%)		
Moderate	21 (32.8)	18 (28.1)
High	43 (67.2)	46 (71.9)
Final statin dose, n (%)		
5 mg	-	7 (10.9)
10 mg	8 (12.5)	9 (14.1)
15 mg	-	1 (1.6)
20 mg	12 (18.8)	8 (12.5)
30 mg	-	34 (53.1)
40 mg	10 (15.6)	5 (7.8)
60 mg	28 (43.8)	-
80 mg	6 (9.4)	-
Final treatment intensity, n (%)		
Moderate	17 (26.6)	14 (21.9)
High	47 (73.4)	50 (78.1)
Dose escalation (yes), n (%)	46 (71.9)	51 (79.7)
Number of dose-escalation steps, n (%)		
0	18 (28.1)	13 (20.3)
1	20 (31.3)	22 (34.4)
2	25 (39.1)	28 (43.8)
3	1 (1.6)	1 (1.6)

For the purposes of this analysis, atorvastatin doses of 10–20 mg and rosuvastatin doses of 5–10 mg were classified as moderate-intensity therapy, whereas atorvastatin doses ≥30 mg and rosuvastatin doses ≥15 mg were classified as high-intensity therapy. Dose escalation was defined as at least one increase in statin dose during follow-up. The number of dose escalation steps represents the number of increases in statin dose between consecutive treatment intervals.

Supplementary Table S2. Distribution of statin doses across consecutive 4-week treatment intervals according to treatment group.

Treatment characteristics	Atorvastatin, n (%)	Rosuvastatin, n (%)
Weeks 0–4 (n = 64 vs. n = 64)		
5 mg	-	18 (28.1)
10 mg	21 (32.8)	-
20 mg	-	45 (70.3)
30 mg	-	1 (1.6)
40 mg	43 (67.2)	
Weeks 4–8 (n = 61 vs. n = 63)		
5 mg	-	7 (11.1)
10 mg	7 (11.5)	11 (17.5)
20 mg	12 (19.7)	5 (7.9)
30 mg	-	39 (61.9)
40 mg	8 (13.1)	1 (1.6)
60 mg	34 (56.0)	-
Weeks 8–12 (n = 36 vs. n = 41)		
5 mg	-	2 (4.9)
10 mg	3 (8.3)	3 (7.3)
15 mg	-	3 (7.3)
20 mg	2 (5.6)	2 (4.9)
30 mg	4 (11.1)	5 (12.2)
40 mg	2 (5.6)	26 (63.4)
60 mg	3 (8.3)	-
80 mg	22 (6.1)	-
Weeks 12–16 (n = 10 vs. n = 13)		
5 mg	-	1 (7.7)
10 mg	-	5 (38.5)
15 mg	-	1 (7.7)
20 mg	2 (20.0)	2 (15.4)
40 mg	1 (10.0)	4 (30.8)
60 mg	1 (10.0)	-
80 mg	6 (60.0)	-

Statin doses were recorded in consecutive 4 week treatment intervals. Percentages were calculated using the number of participants receiving treatment during the respective interval as the denominator. The number of participants decreased across treatment intervals because final biomarker measurements were performed at different follow up times.

Supplementary Table S3. Adjusted linear regression analyses of final endocan and endoglin concentrations according to statin treatment.

Predictor	Final endocan B (95% CI)	*P* value	Final endoglin B (95% CI)	*P* value
†Statin type	−51.24 (−342.30 to 239.81)	0.21	100.03 (−55.18 to 255.44)	0.20
‡Baseline biomarker	−0.738 (−0.782 to −0.693)	**<0.001**	0.27 (0.19 to 0.35)	**<0.001**
Baseline LDL-C	−12.38 (−323.30 to 298.53)	0.64	−83.42 (−172.31 to 5.46)	0.07
Baseline triglycerides	−222.49 (−651.43 to 206.45)	0.97	−53.50 (−153.16 to 46.17)	0.29
Baseline GGT	0.03 (−10.02 to 10.08)	0.17	2.32 (−2.59 to 7.23)	0.35
Model statistics	R^2 = 0.557; adjusted R^2 = 0.538; $P < 0.001$		R^2= 0.315; adjusted R^2 = 0.287; $P < 0.001$	

Values are unstandardized regression coefficients (B) with 95% confidence intervals (CI). LDL-C = low-density lipoprotein cholesterol; GGT = gamma-glutamyl transferase. †Statin treatment group was coded as 1 = atorvastatin and 2 = rosuvastatin. ‡Baseline corresponding biomarker refers to baseline endocan in the model for final endocan and baseline endoglin in the model for final endoglin. Bold values indicate statistical significance.
